# Exposure to cigarette smoke precipitates simple hepatosteatosis to NASH in high-fat diet fed mice by inducing oxidative stress

**DOI:** 10.1042/CS20210628

**Published:** 2021-09-06

**Authors:** Sherouk Fouda, Anwar Khan, Stanley M.H. Chan, Ali Mahzari, Xiu Zhou, Cheng Xue Qin, Ross Vlahos, Ji-Ming Ye

**Affiliations:** 1 School of Health and Biomedical Sciences, RMIT University, Melbourne, VIC, Australia; 2 Faculty of Pharmacy and Pharmaceutical Sciences, Monash University, VIC, Australia; 3 Department of Laboratory Medicine, Faculty of Applied Medical Sciences, Albaha University, Albaha 65527, Saudi Arabia

**Keywords:** cigarette smoking, High fat diet, non-alcoholic steatohepatitis, oxidative stress

## Abstract

Consumption of diet rich in fat and cigarette smoking (CS) are independent risk factors of non-alcoholic steatohepatitis (NASH), and they often occur together in some populations. The present study investigated the mechanisms of high-fat diet (HFD) and CS, individually and in combination, on the pathogenesis of NASH in mice. C57BL/6 male mice were subjected to either a low-fat chow (CH) or HFD with or without mainstream CS-exposure (4 cigarettes/day, 5 days/ week for 14 weeks). HFD alone caused hepatosteatosis (2.5-fold increase in TG content) and a significant increase in 3-nitrotyrisine (by ∼40-fold) but without an indication of liver injury, inflammation or fibrosis. CS alone in CH-fed mice increased in *Tnfα* expression and macrophage infiltration by 2-fold and relatively less increase in 3-nitrotyrosine (18-fold). Combination of HFD and CS precipitated hepatosteatosis to NASH reflected by exacerbated makers of liver inflammation and fibrosis which were associated with much severe liver oxidative stress (90-fold increase in 3-nitrotyrisine along with 6-fold increase in carbonylated proteins and 56% increase in lipid oxidations). Further studies were performed to administer the antioxidant tempol to CS exposed HFD mice and the results showed that the inhibition of liver oxidative stress prevented inflammatory and fibrotic changes in liver despite persisting hepatosteatosis. Our findings suggest that oxidative stress is a key mechanism underlying CS-promoted progression of simple hepatosteatosis to NASH. Targeting hepatic oxidative stress may be a viable strategy in halting the progression of metabolic associated fatty liver disease.

## Introduction

The escalating impact of non-alcoholic fatty liver disease (NAFLD), currently recognized as metabolic associated fatty liver disease (MAFLD) together with its progressive stage, non-alcoholic steatohepatitis (NASH) on global health has attracted much attention in recent years [1]. NASH is a critical advanced stage of MAFLD where its prognosis is dramatically deteriorated from a reversible to an irreversible stage with significant increases in liver cirrhosis and cancer [2,3]. The current global prevalence of MAFLD stands at nearly 25 percent and it is highly associated with hyperlipidemia (69%), obesity (51%), metabolic syndrome (43%) and type 2 diabetes (22%) among other metabolic comorbidities [4]. MAFLD usually starts as fatty liver without other associated pathological changes (namely simple hepatosteatosis, short as hepatosteatosis here after) not related to alcohol consumption or other toxic effects [3]. While hepatosteatosis by itself is relatively ‘benign’, approximately 30% progresses to NASH which includes hepatic inflammation, hepatic injury and various degrees of hepatic fibrosis [5].

One of the most common causes of hepatosteatosis is the consumption of diet rich in fat, which increases influx of diet-derived fatty acids into the liver. The mechanisms involved in high-fat diet (HFD) induced hepatosteatosis have been well described in studies using rodent models. HFD causes the metabolic syndrome including obesity, fasting hyperglycaemia, hyperinsulinemia, and lipid accumulation in the liver, similar to the phenotype observed in humans with MAFLD. Despite severe hepatosteatosis, it is clear now that HFD *per se* is not sufficient to result in NASH even after a prolonged period of feeding [6,7] and even after 80 weeks of chronic feeding [8]. This is consistent with the findings in humans that NASH results from the interplay of multiple mechanisms centred around the metabolic syndrome [3,9]. It is likely these multiple mechanisms result from heterogenous causes or ‘hits’ in different populations under the heavy influences of various lifestyle factors.

One such unhealthy lifestyle factor is cigarette smoking (CS) which still occurs in a considerable large population. Recent epidemiological studies suggest that exposure to CS is associated with exacerbated severity of MAFLD at various stages [10,11]. Intriguingly, CS has been shown to lessen body weight gain, and arguably this may help reduce hepatosteatosis, thus alleviating NASH. However, the consequence of and mechanisms involved in the interplay of these two co-existing lifestyle factors have not been carefully investigated in relation to the progress of MAFLD.

It is well established that CS is associated with the stable free radicals presence, particularly in the tar phase of the smoke [12]. These oxidants grant CS the capacity to oxidize proteins, lipids and DNA to damage to organs, which may potentially result in hepatocyte injury, inflammation and even fibrosis. Additionally, CS may promote lipolysis in adipose tissue, thereby increasing free fatty acids in the circulation and their ectopic storage into non-adipose tissues such as the liver [13,14], particularly during obesity where the storage capacity of adipose tissue may be overwhelmed [15,16]. In the present study, we sought to address whether CS exposure accelerates the development of MAFLD in the context of HFD feeding-induced obesity, and the role of oxidative stress as an underlying mechanism. We hypothesize that CS induced oxidative stress has additive or even synergistic effect with HFD to drive the progression of MALFD toward NASH. If so, inhibition of oxidative stress should negate, at least in parts, the deleterious effects of CS-exposure on MAFLD.

## Material and methods

### Animals and experimental design

Male C57BL/6J mice (10 weeks old) were acquired from the Animal Resource Centre, Perth, Australia. All experiments were approved by the RMIT University Animal Ethics Committee (#1705) and performed in compliance with the National Health and Medical Research Council (NHMRC) of Australia guidelines. All experiments were performed at the RMIT University Animal Facility. They were kept at 22 ± 1°C on a 12 h light/dark cycle. After 2 weeks of acclimatization, animals were randomly subdivided into respective groups. Mice were fed *ad libitum* for 14 weeks either a chow (CH) consisting crude protein (21% kcal), fat (8% kcal), and carbohydrates (71% kcal) and supplies total metabolic energy of 3.34 kcal/g, or a HFD containing 25% fat supplying energy (45% kcal) from fat of which 40% kcal comes from saturated fats (lard), protein (20% kcal), and carbohydrates from a mix of starch, sucrose and bran (35% kcal) and having a total metabolic energy of 4.938 kcal/g. Diets were stored at −20°C for less than one month and were changed daily during the experiments.

For the terminal studies, mice were then culled by cervical dislocation. Liver tissues were harvested, and left lobe was fixed in 10% neutral buffered formalin for histological analysis and the rest was snap-frozen in liquid nitrogen prior to −80°C storage for further analysis. Blood was collected directly from the heart. The samples were then centrifuged at 10,000 rpm for phase separation, and the plasma was immediately stored at −80°C for future assays.

### Cigarette smoke exposure and tempol treatment

Mice on CH or HFD were chronically exposed to CS generated from 4 cigarettes/day (2 sessions of 2 cigarettes), 5 days/ week for 14 weeks. Age-matched control mice were exposed to room air (sham). Mice were placed in an 18-liter perspex chamber and exposed to CS generated from four Winfield Red cigarettes per day (<16 mg tar, <1.2 mg nicotine, <15 mg CO; Philip Morris, Moorabbin, Australia) intermittently to mimic the behaviour of human smokers, 5 days per week, or air and weighed twice weekly. At the end of 14 weeks, mice were killed by cervical dislocation. Group sizes of 9 to 10 mice per treatment were used. One group of mice who received a combined HFD and smoking (HFD-S) were treated with 4-hydroxy-2,2,6,6-tetramethylpiperidine-N-oxyl (tempol) to test the hypothesis that the antioxidant tempol attenuates the progression to NASH by blocking oxidative stress. Tempol was dissolved in the drinking water at a final concentration of 1 mM. Daily fluid consumption was monitored throughout the 14-week treatment period.

### Body weight, food intake and blood /plasma measurements

Body weight and food intake were monitored twice weekly throughout the experiment. For blood and plasma collection, mice were fasted 5–7 h prior to tail vein blood being collected for further analysis. At the end of the study, blood was collected from the right ventricle using cardiac puncture, in ethylenediaminetetraacetic acid (EDTA) coated tubes then centrifuged at 1,500 *** g*** for 1 min and plasma was stored at −80°C for biochemical assays.

Glucose tolerance test (GTT) (glucose 1.5 g/kg, i.p.) was performed after 5–7 h of fasting in week 12 using a glucometer (Accu-Chek; Australia). Blood glucose levels were measured from the tail tip at 0, 15, 30, 60, and 90 min using glucometer. Insulin tolerance test (ITT) was performed after 5–7 h of fasting in week 13. Blood glucose levels were measured from the tail tip at 0, 20, 40, 60, 80, and 120 min.

Plasma alanine aminotransferase (ALT) was measured using commercial kits (ALT/SGPT Liqui-UV Kit, Boerne, U.S.A.) [6]. Animals were fasted for 5–7 h, blood plasma was collected from the tail vein and prepared according to the manufacturer’s instructions. The absorbance was measured at 340 nm using a FlexStation (Molecular Devices, Sunnyvale, CA, U.S.A.). Plasma levels of total triglycerides (TG) and free fatty acids (FFA) were determined using Triglyceride GPO-PAP and NEFA kits (Roche Diagnostics, Australia).

### Histological analysis of liver

Liver tissues stored in 10% neutral buffered formalin were dehydrated using a Leica tissue processor (Leica, Australia). The dehydrated samples were embedded in paraffin and cut into 4-μm-thick sections. Mayer’s Hematoxylin and Eosin (H&E) staining was performed and images were taken from Olympus BX41 microscope with a 20 times objective lens using an Olympus DP72 digital camera (Olympus, Australia). Paraffin-embedded sections were stained with picrosirius red stain to visualize collagen I and III fibers. Slides were quantified by randomly selecting five non-overlapping fields of view per slide representing one mouse using an Olympus BX41 microscope with a 20× objective lens and an Olympus DP72 digital camera (Olympus, Australia). The five regions of interest (ROIs) were selected on the liver slides, with care taken to avoid large hepatic vessels or artifacts. Sections were blinded and scored by two independent observers (*n* = 8–10). The mean of value was calculated for each experimental group using the threshold function in the ImageJ software package (NIH Image, Bethesda, MD, United States). Data are represented as relative fold change to CH group.

### Immunohistochemistry/Immunofluorescence

Liver sections were deparaffinized, hydrated followed by antigen retrieval by heat, then sections were blocked and incubated overnight at 4°C with primary antibody anti-F4/80 (1:100) diluted in PBS-T containing 5% fetal bovine serum (FBS). Sections were subsequently washed three times and incubated for 1 h at room temperature with streptavidin APC secondary antibody, and 1-h incubation in avidin-biotin horseradish peroxidase (HRP) complex (VECTASTAIN® Elite® ABC-HRP Kit, Vector Laboratories Ltd). After development in with 3,3′-diaminobenzidine tetrachloride (DAB) solution, sections were counter stained with haematoxylin. Images were acquired using VS120 Virtual Slide Microscope and analyzed using the CELLSENS™ life science imaging software (Olympus, Australia). For quantification F4/80-positive hepatic macrophages in liver section, five random non-overlapping selected fields of view per slide at ×200 magnifications were examined and determined for eight animals in each group.

Liver oxidative stress was measured using 3-nitrotyrosine (3-NT) (1:100 dilution, Thermo Fisher Scientific, USA) as this is a specific marker for peroxynitrite production, which is a result of superoxide and nitric oxide interaction. Liver sections were subject to standard deparaffinisation and rehydration, followed by antigen retrieval (in 10 mM citric acid, 0.05% Tween 20, pH 6.0) and blocking (blocking buffer; 10% horse serum, 10% FBS, 2% triton-X, 1× PBS to 50 ml for 1 h). Sections were then incubated overnight with 3-NT primary antibody at 4°C. Sections were washed, then incubated at room temperature with the fluorescence labelled secondary antibody, Alexa 488 (1:200 dilution, AB_2633275 Thermo Fisher Scientific, U.S.A.), then cover slipped using Fluoromount-GTM, with DAPI (Thermo Fisher Scientific, U.S.A.) prior to imaging on an Olympus slide scanner VS120-SS (Olympus, Japan). The expression of 3-NT was quantified in using Olympus CellSens Dimension TM desktop software, calculating Object Area Fraction ROI (%) and data presented as fold change from CH group (version 1:18, Olympus Corporation). All analysis of immunofluorescence was completed in a blinded manner.

### Enzyme Linked Immunosorbent Assay (ELISA)

ELISA were completed on liver homogenates for COL4A1 protein, using PBS homogenized liver tissues. Liver homogenates supernatant was loaded into LSBio® mouse COL4A1 ELISA kit (LifeSpan Biosciences, U.S.A.) and completed as per manufacturer’s instructions. Assay end-product absorbance was determined spectrophotometrically using a plate reader at 450 nm (CLARIOstar®, BMG LabTech, U.S.A.) and COL4A1 concentration was determined using calculated mean absorbance data from duplicated samples using the standard curve. All data were plotted using GraphPad Prism TM for Microsoft Windows® (Versions 8, Graphpad software®, U.S.A.).

### Western blotting

Western blotting was performed as described previously [17,18]. Proteins prepared in Laemmli buffer were separated by SDS-PAGE, then transferred to PVDF membranes (Bio-Rad, U.S.A.) and blocked in 3% BSA. Membranes were probed with the following primary antibodies; glyceraldehyde 3-phosphate dehydrogenase (GADPH), nuclear factor κβ (NFκβ), inhibitor of κβ (Iκβ) antibodies were purchased from Cell Signaling (U.S.A.). Secondary Goat Anti-Rabbit antibody conjugated to horse radish peroxidase (#sc-2004) was purchased from Santa Cruz (U.S.A.). The membranes were developed using enhanced chemiluminescence HRP substrate reagents (Perkin Elmer, U.S.A.). Images of the membranes were taken with the ChemiDoc system and densitometry analysis was performed using Image Lab software (Bio-Rad Laboratories, U.S.A.).

### Quantitative real-time PCR

RNA was extracted using RNeasy Kit (Qiagen), while RNA extract was reverse-transcribed using a High capacity cDNA reverse transcription kit (Life Technologies, Australia) according to the manufacturer’s instructions. Bioinformatically validated Taqman Primers (Life Technologies, Australia) including glyceraldehydephosphate dehydrogenase (*Gapdh*, Mm03302249 g1), IL-6 (*Il6*, Mm00446190 m1), IL-1β (*Il1β*, Mm00434228 m1), TNFα (*Tnfα*, Mm004432458 m1), TGFβ (*Tgfb1*, Mm01178820 m1), Col1 (*Col1a1*, Mm00801666 g1), α-SMA (*Actin 2*, Mm00725412 s1), NOX2 (*Cybb*, Mm01287743 m1), NOX4 (*Nox4*, Mm00479246 m1), HMOX1 (*HO-1*, Mm00516005 m1), CD68 (*Cd68*, Mm03047343 m1), glutathione peroxidase (*Gpx1*, Mm00656767 g1).

All reactions were performed at 50°C for 2 min, 95°C for 3 min, 40 cycles of 95°C for 15 s, 72°C for 30 s and followed by measurements of melt curve using QIAGEN Rotor-Gene Q PCR system (Germany). All reactions were performed in triplicates and GAPDH was used as the normalizing control gene and results were analyzed by the ΔΔCt method.

### Characterization of the phenotypes of MAFLD/NASH

#### Hepatic steatosis: liver TG content measurement

Lipids were extracted from the freeze-clamped liver tissue by the method of Bligh and Dyer [19]. TG levels were quantifiably determined by an enzymatic method specific for TG using a TG GPO-PAP kit (Roche Diagnostics, Australia). A representative image hepatic steatosis in H&E staining for each group was also supplemented.

#### Evaluation of hepatic inflammation, injury and fibrosis

Hepatic inflammation was evaluated by RT-PCR of pro-inflammatory markers (*Tnfα*, *Il1β, Il6*, *Cd68)* and immunohistochemical staining of F4/80 (BM8, Fisher Scientific, U.S.A.), a unique marker of murine macrophages.

For hepatic injury, plasma alanine aminotransferase (ALT) levels were measured in week 14 using commercial kit (ALT/SGPT Liqui-UV Kit, Boerne, U.S.A.). Whole blood was collected from 5 to 7 h fasted mice via tail vein and processed according to the manufacturer’s instructions. The absorbance was measured at 340 nm using a FlexStation (Molecular Devices, Sunnyvale, CA, U.S.A.).

Hepatic fibrosis was confirmed by RT-PCR of fibrogenic markers, including *Tgfb1, Col1a1* and *Actin2* gene expressions by real time PCR analysis using QuantStudio 7™ (Applied Biosystem, U.S.A.).

#### Measurements of oxidative stress

Malondialdehyde (MDA) was measured as a marker of systemic oxidative stress. Liver MDA levels were determined using commercial kit OxiSelect TBARS Assay Kit (MDA Quantitation) from Cell Biolabs (#STA-330). The TBARS (Thiobarbituric Acid Reactive Substances) assay was used to quantify lipid peroxidation in liver homogenates. liver homogenates were prepared according to manufacturer’s instructions and MDA levels were normalised to protein content.

As described above, 3-NT levels were determined using immunofluorescent staining technique. Briefly, sections were antigen retrieved and incubated with diluted primary antibody 3-NT (1:200) in blocking buffer overnight at 4°C. Sections were then washed with PBS-T and incubated with secondary diluted 1:200 in blocking buffer, for 1 h at room temperature in a humidifier and treated as photosensitive in all subsequent stages. Finally, sections were washed and mounted using Fluoroshield TM DAPI, histological mounting medium (Sigma-Aldrich, USA) and stored until quantification. The expression of 3-NT was quantified in the left lobe of liver using Olympus CellSens DimensionTM desktop software, calculating Object Area Fraction ROI (%) and data presented as relative fold change to CH (version 1:18, Olympus Corporation).

### Statistical analyses

All data were expressed as mean ± SEM, where each *n* represents one mouse unless otherwise indicated. Where applicable, one- or two-way ANOVA with Tukey or Bonferroni’s *post hoc* tests were used. Statistical analysis was performed using Prism GraphPad software version 5.0c (Software MacKiev, San Diego, CA), with *P*<0.05 accepted as being statistically significant.

## Results

### Effects on body weight and whole-body metabolism

**Figure 1:**
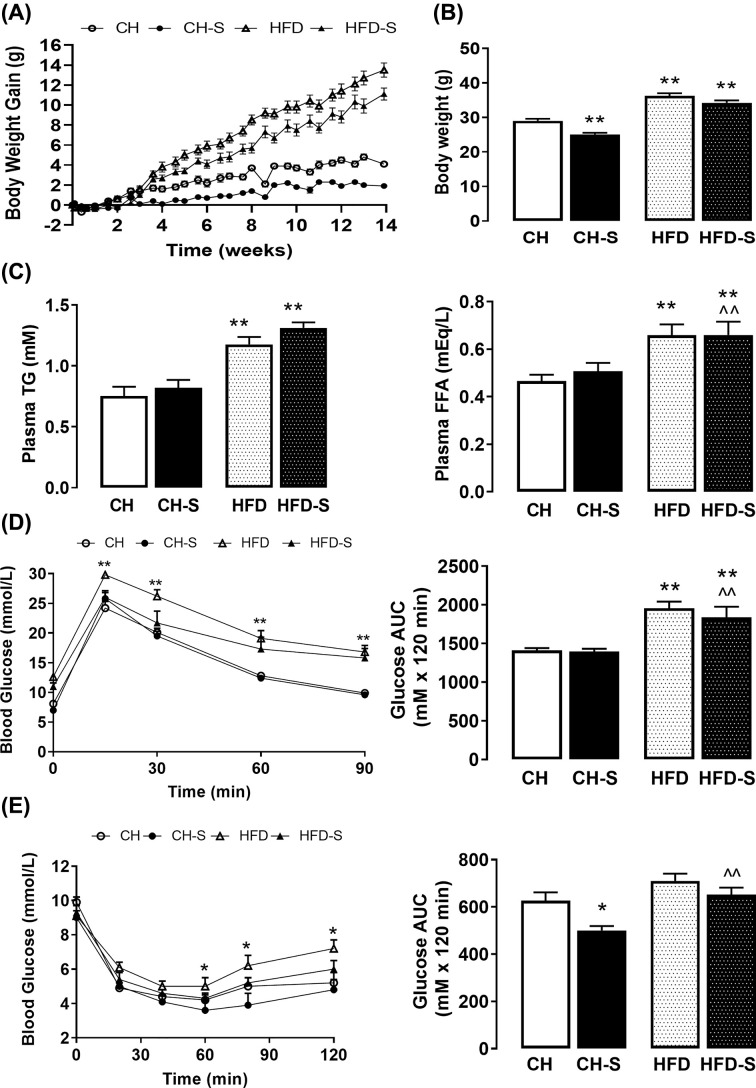
Effects of smoking on the body weight and whole-body metabolism Accumulative changes in body weight over time (**A**). Low fat normal chow-fed diet alone (CH, ○), high-fat diet alone (HFD, Δ), chow diet fed mice exposed to CS (CH–S, ●), and high-fat diet fed mice exposed to CS (HFD–S, ▲) (**B**) End-point body weight. (**C**) Plasma levels of triglycerides (TG) and free fatty acids (FFA). (**D**) Glucose tolerance test (GTT) assessed after 12 weeks and blood glucose area under the curve (AUC) analysis of the GTT. (**E**) Insulin tolerance test (ITT) assessed after 14 weeks and blood glucose AUC analysis of the ITT. Results are expressed as means + SEM (*n* = 8–10/group). Statistical analyses were performed by two-way ANOVA with repeated measures followed by Bonferroni post hoc test; *P<0.05, **P<0.01 vs. CH group and ^⁁^*P*<0.05, ^⁁⁁^*P*<0.01 vs. CH-S.

Body weight of all groups was well matched at the beginning of the experiment (Figure 1A). High fat feeding resulted in a 25% increase in body weight (*P*<0.01) and a 60% increase in fasting plasma glucose levels compared to the CH group (Figure 1D). Accumulated food intake per group showed a reduction of 12% in CH-S (CH, 246.9 g; CH-S, 216.6 g) and less than 10% in the HFD-S (HFD, 244.9 g to 221.5 g) groups compared with the corresponding non-smoking groups. The combination of CS and high-fat diet also resulted in a significant body weight increase of 26% compared with chow group (Figure 1B). As expected, HFD resulted in a significant increase in circulating levels of TG and FFA, regardless of smoking status (Figure 1C). When glucose challenged, mice on HFD displayed significant glucose intolerance evidenced by a ∼25% increase in AUC, which was not significantly worsened by CS (Figure 1D). HFD alone resulted in no significant changes in insulin tolerance compared with CH group. To our surprise, CS resulted in a marked improvement of over 20% in insulin tolerance in the CH group but not the HFD group (Figure 1E).

### Effects on hepatic steatosis

**Figure 2:**
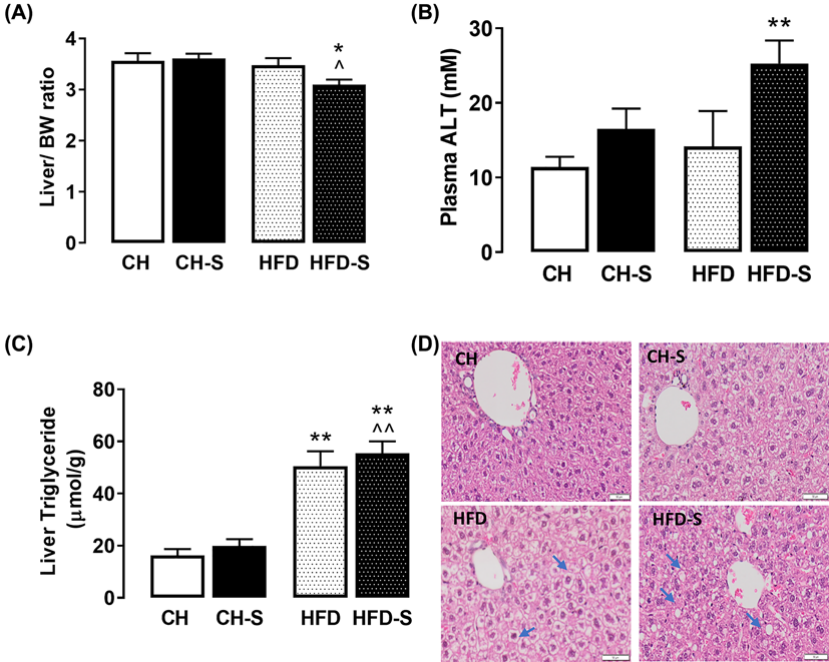
Effects of smoking on hepatic weight, enzyme and triglyceride content (**A**) Liver weight expressed as a ratio to the body weight (BW), (**B**) Plasma levels of alanine aminotransferase (ALT, indicative of liver injury), and (**C**) liver triglyceride (TG) content normalized against differences in protein concentration. (**D**) Representative images of H&E staining of liver sections from chow (CH), chow smoke exposed (CH-S), high-fat diet (HFD) and high-fat diet smoke exposed (HFD-S). Scale bar represents 50 μM. Arrows indicate the shape of lipid droplets. Data are expressed as mean + SEM and analyzed by two-way ANOVA with Tukey’s multiple comparisons, *n*=8–10. *P<0.05, **P<0.01 vs. CH group, ^⁁^*P*<0.05, ^⁁⁁^*P*<0.01 vs. CH-S group.

In line with our previous findings [6], HFD increased the accumulation of TG in the liver by 2-fold, but this did not result in any detectable alterations in liver mass (Figure 2A). Despite a more moderate weight gain, the combination of HFD and CS caused a ∼15% reduction in liver mass (Figure 2A) which was associated with a significant increase in plasma ALT (Figure 2B), suggesting possible liver injury in these mice. Meanwhile, CS did not exacerbate the hepatic TG accumulation caused by HFD (Figure 2C). In line with the biochemically quantified hepatic steatosis, Figure 2D showed the representative H&E staining for each of these groups.

### Effects on hepatic inflammation and macrophage infiltration

**Figure 3:**
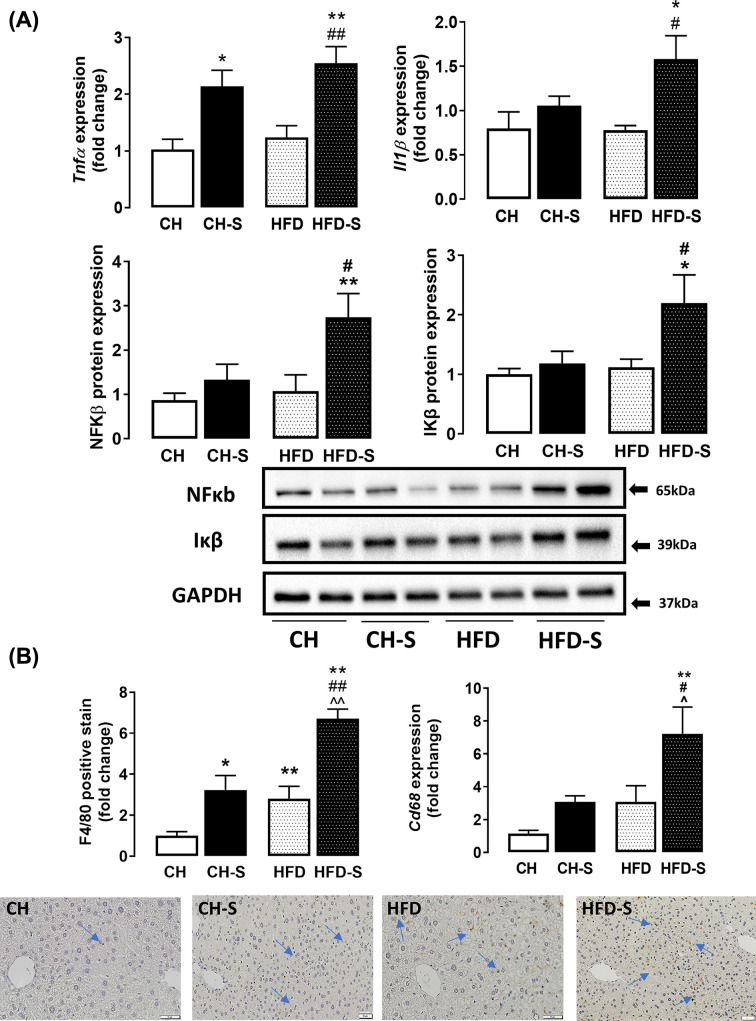
Effects of smoking on hepatic inflammation and macrophage content Whole liver lobes excised from mice were used to measure (**A**) mRNA expression of *Tnfα* and *Il1β*, or protein levels of NFκB and Iκβ by immunoblotting, and GAPDH was used as loading control. (**B**) CS effects on macrophage infiltration in liver was evaluated by immunohistochemistry (IHC) staining of F4/80 and quantified using CellSens software. Representative images of F4/80 stained liver sections (20× magnification) are shown. Scale bar represents 50 μM. The expression of the Kupffer cell specific surface marker, CD68 was analyzed using qPCR. Data are expressed as mean fold change from CH + SEM and analyzed by two-way ANOVA with Tukey’s multiple comparison, *n*=8; *P<0.05, **P<0.01 vs. CH group; ^⁁^*P*<0.05, ^⁁⁁^*P*<0.01 vs. CH-S group; ^#^*P*<0.05, ^##^*P*<0.05 vs. HFD group.

To further investigate the effect of CS exposure on HFD-induced liver pathology, we examined key biomarkers and histological features of hepatic inflammation. In the HFD group, CS resulted in a marked induction of pro-inflammatory cytokine mRNA (*Tnfα* and *Il1β*) of ∼2.3- and ∼1.5-fold respectively and protein expression of NFκB and IκB (Figure 3A). F4/80+ staining was increased by ∼2.5-fold by CS or HFD alone, and this was further potentiated to ∼6-fold when the two were combined (Figure 3B). Likewise, *Cd68* mRNA expression also displayed an up-regulation of ∼7-fold in response to by the combination of CS and HFD (Figure 3B). Together, these findings suggest the pro-inflammatory effects of CS and HFD in the liver which may promote the development of fibrosis, a key feature of NASH. Macrophage infiltration in the liver was also assessed using F4/80 macrophage marker using immunohistochemistry. CS alone and HFD alone increased F4/80 expression by ∼3-fold and the combination of CS and HFD markedly increased F4/80 macrophage expression by ∼ 7-fold.

### Effects on hepatic fibrosis and the role of oxidative stress

**Figure 4:**
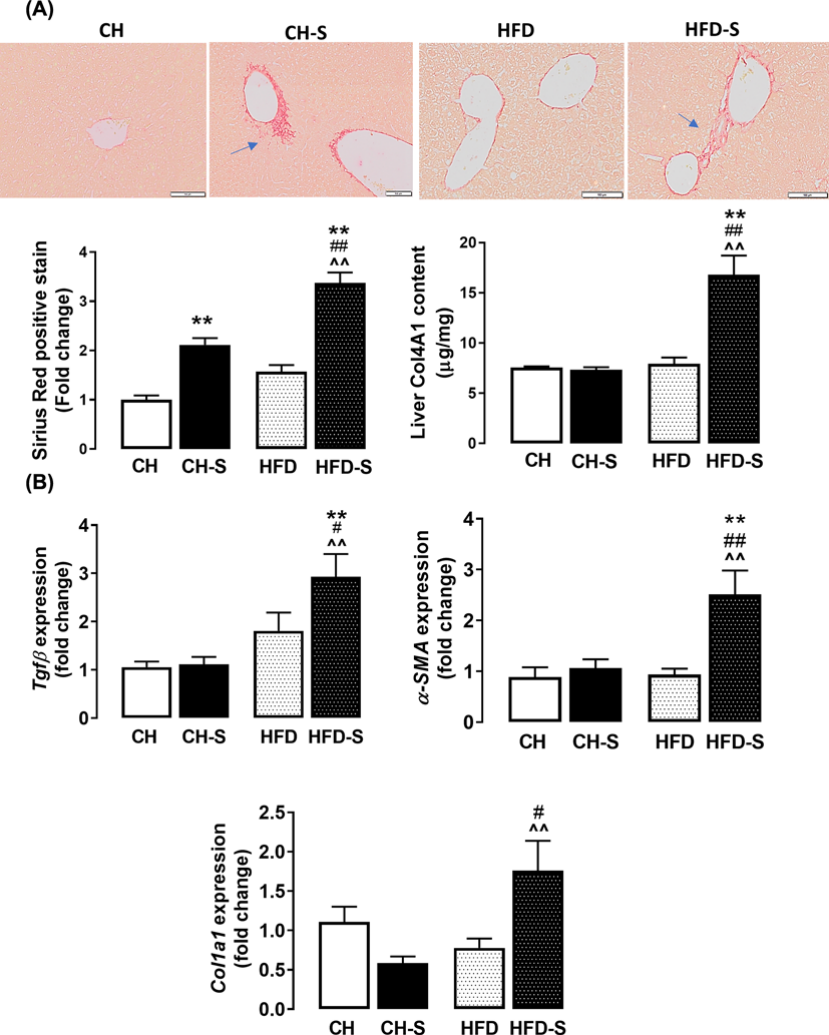
Effects of smoking on hepatic fibrosis Picro sirius red stain was used to stain collagen fibres in liver sections (4 µm). (**A**) Representative images showing collagen positive stains (20× magnification). Scale bar represents 50 µm. Arrows indicate the presence of collagen fibres. After normalization to the relevant negative control, positive staining was quantified and expressed as fold change from the CH group (*n*=8–10). The total content of collagen 4A1 in the liver was determined by immunosorbent assay. Data were normalized to protein concentration. (**B**) mRNA expression of *Tgfβ*, *αSma* and *Col1α*. Data are expressed as mean + SEM and analyzed by two-way ANOVA with Tukey’s multiple comparisons, *n*=8–10. **P<0.01 vs. CH group, ^##^*P*<0.01 vs. HFD group, ^⁁⁁^*P*<0.01 vs. CH-S group.

**Figure 5:**
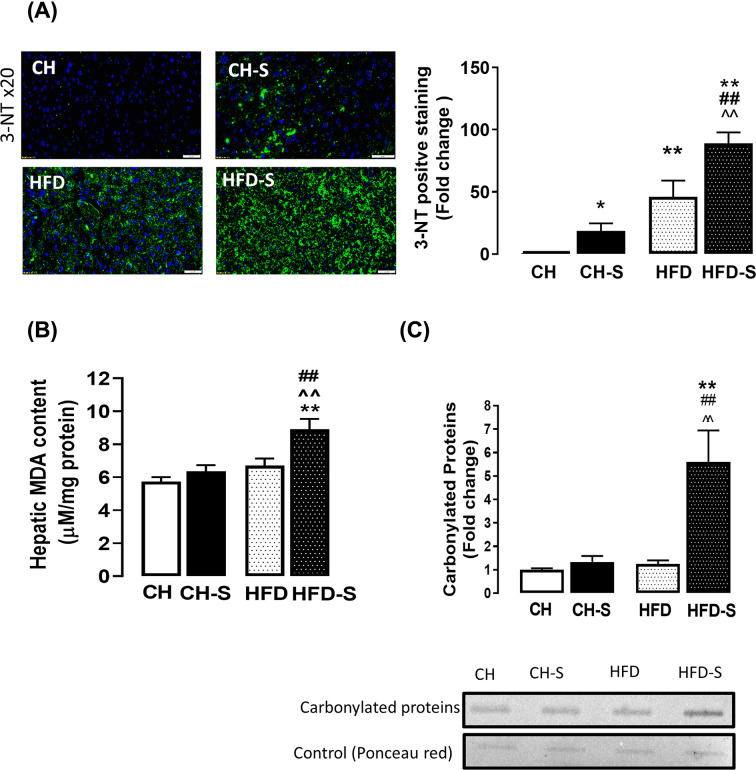
Effects of smoking on oxidative stress in the liver (**A**) Representative images of 3-NT (green) immunostaining of liver sections (20× magnification); nuclei are counterstained with DAPI (blue). Scale bar represents 50 µm. After normalization to the relevant negative control, the 3-NT positive staining was quantified and presented as fold change from CH group. (**B**) Lipid peroxidation was assessed by measuring MDA concentration in the liver. (**C**) The degree of protein oxidation in the liver was determined by Oxyblot assay. Data are expressed as mean + SEM and analyzed by two-way ANOVA with Tukey's multiple comparisons, *n*=8–10; *P<0.05, **P<0.01 vs. CH group, ^##^*P*<0.01 vs. HFD group, ^⁁⁁^*P*<0.01 vs. CH-S group.

**Figure 6:**
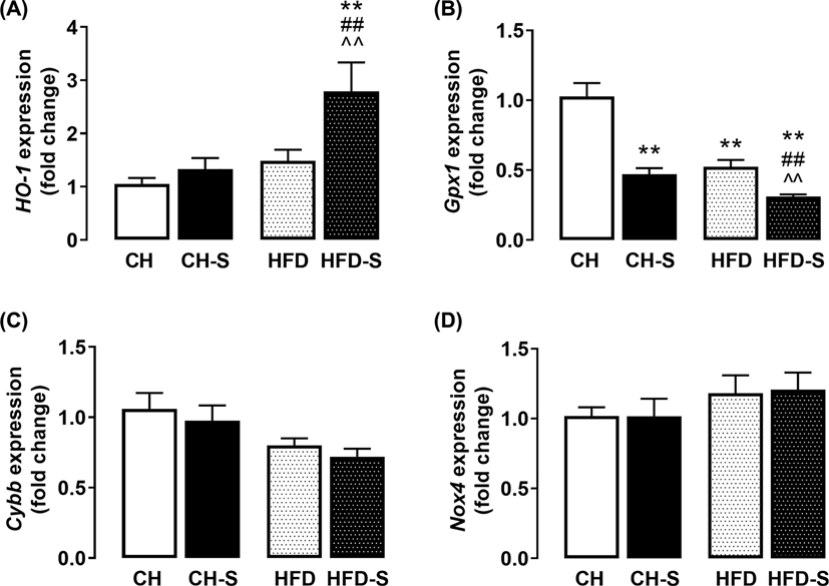
Effects of smoking on redox enzymes in the liver mRNA expression of key hepatocellular antioxidants (**A**) *HO-1* and (**B**) *Gpx1*, and oxidant generating enzymes including (**C**) *Cybb* (Nox2) and (**D**) *Nox4*. Data are expressed as fold change relative to CH. Data are expressed as mean + SEM and analyzed by two-way ANOVA with Tukey's multiple comparisons, n = 8-10. ** P< 0.01 vs CH group, ## P < 0.01 vs HFD group, ⁁⁁ P < 0.01 vs CH-S group.

Sirius red staining revealed that CS, but not HFD alone, increases collagen deposition and this effect was potentiated when the two were combined (Figure 4A). In line with this, the mRNA expression of *Tgfβ*, *Acta2* and *ColIa* were also up-regulated (∼3-, 2.5-, and 1.5-fold respectively) by the combination of CS and HFD (Figure 4B), confirming the presence of hepatic injury and fibrosis. Collagen 4 in the liver was measured biochemically and a significant increase (∼2-fold) was shown in the combined group HFD-S compared with CH. As liver injury has been suggested to be most commonly associated with an imbalance intracellular redox environment which may result in oxidative stress [20], we reasoned whether this may underlie the CS-induced hepatic injury and fibrosis. Our immunohistochemical analysis revealed that the hepatic levels of 3-nitrotyrosine (3NT) were significantly augmented by HFD (50-fold) and this was further exacerbated by CS (90-fold; Figure 5A). In line with this, the hepatic level of MDA, a product of oxidative damage to membrane lipids [21], was found to be elevated (56%) in the HFD-S group; MDA concentrations were 5.7 ± 0.9 µM/mg, 6.4 ± 1.2 µM/mg, 6.7 ± 1.2 µM/mg, and 8.9 ± 1.8 µM/mg for the CH, CH-S, HFD, and HFD-S groups, respectively (Figure 5B). In addition to lipids, protein carbonylation, a result of protein oxidation, was also found to be significantly increased (∼5-fold) by HFD-S (Figure 5C), suggesting the CS-induced hepatic injury and fibrosis during the feeding of HFD is likely to be related to the induction of oxidative stress within the tissue. Indeed, our qPCR analysis found that the combination of CS and HFD resulted in a significant increase (∼2-fold) in the mRNA expression of *HO-1* and a concomitant suppression of *Gpx1* (Figure 6A,B), which are key antioxidant enzymes that protects against cellular oxidative stress [22]. Of note, the mRNA expression of *Cybb* (encodes Nox2) and *Nox4* (Figure 6C,D), two major oxidant generating enzymes, remained unaltered regardless of smoking or HFD. These findings not only confirmed the presence of oxidative stress within the tissue but also raise an important notion that the origin of this CS-induced oxidative stress is unlikely to be endogenous in origin.

### Effects of inhibiting hepatic oxidative stress on NASH induced by HFD and CS

**Figure 7:**
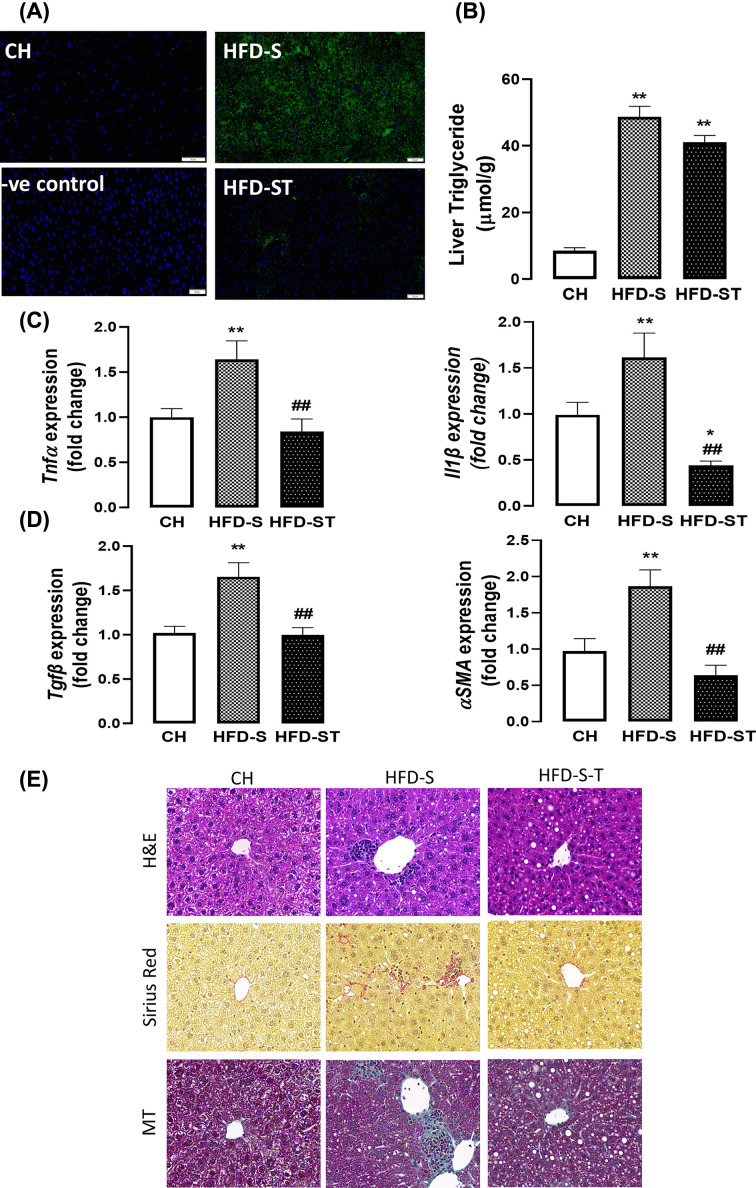
Inhibition of oxidative stress on smoking-induced NASH Mice were fed the HFD as described for 14 weeks and exposed to cigarette smoking (twice daily/2CS/ session) and maintained from week 0 to 14, with or without the oral administration of tempol in drinking water (1 mM). (**A**) Liver triglyceride (TG) content per wet tissue weight (**B**) Representative images of staining with 3-nitrotyrisine (3NT) expression in liver (green), while nuclei are counterstained with DAPI (blue). After normalisation to the relevant negative control, the expression of 3-NT is presented as fold change from CH group. Scale bar represents 50 µm. Quantitative PCR was performed to assess the expression of pro-inflammatory markers in the liver; mRNA expression of key fibrogenic markers (**C**) *Tnfα* and *Il1β*, (**D**) *Tgfβ* and *αSma*. Gene expression data are expressed as fold change relative to the CH group. (**E**) Representative images of H&E staining, sirius red staining and masson’s trichrome (MT) staining of liver sections (20× magnification). Scale bar represents 50 μM. Quantitative data are expressed as mean + SEM and were analyzed by one-way ANOVA with Tukey’s multiple comparisons, *n*=8–10.*P<0.05, **P<0.01 vs. CH group, ##*P*<0.01 vs. HFD group.

The strong indication that oxidative stress as an underlying trigger for the hepatic injury have led us to reason whether inhibition of oxidative stress may prevent the deleterious effects of CS and HFD. Tempol administration effectively attenuated the induction of oxidative stress by CS and HFD (HFD-S) evidenced by the decreased 3-NT positive staining in the liver (Figure 7A). Despite the persisting steatosis (Figure 7B), tempol administration completely prevented the induction of key pro-inflammatory (*Tnfα* and *Il-1β*) and fibrogenic (*Tgfβ* and *αSMA*) markers (Figure 7C,D), suggesting a liver protecting effect. Our histopathological analyses revealed that tempol administration largely prevented hepatic collagen accrual and the consequential fibrosis, with no other observable improvements in liver morphology as shown by the H&E staining (Figure 7E). This suggest that inhibition of oxidative stress was effective in preventing the development of NASH but not ectopic lipid accumulation in the liver.

## Discussion

The present study investigated the consequence of and mechanisms involved in the interplay of HFD and CS in relation to the progress from simple hepatosteatosis to NASH. The study was designed to closely mimic that habitual smoking from early life with or without concurrent consumption of diets rich in fat. We found that CS only promoted liver the expression of inflammatory markers and mild fibrosis whereas HFD induced hepatosteatosis and metabolic syndrome phenotypes. Whereas neither CS nor HFD alone resulted in NASH, in combination they induce typical NASH associated with severe hepatic oxidative stress. Furthermore, inhibition of hepatic oxidative stress alleviated most NASH phenotypes produced by the combination of CS and HFD despite persisting steatosis. Together, the present study suggests that oxidative stress is a key mechanism by which CS precipitates simple steatosis to NASH.

Feeding of HFD enriched in long chain fatty acids presents a model of energy surplus that promotes the development of obesity and metabolic syndrome [23]. The dysregulated substrate metabolism in obesity leads to the ectopic accumulation of lipids in the liver, giving rise to hepatic steatosis, which is regarded as the hepatic hallmark of the metabolic syndrome [23]. Hepatic steatosis is characterized by the accumulation TG on over 5% of hepatocytes [24]. This form of steatosis is usually benign in nature and would not proceed to steatohepatitis in the absence of a secondary insult. To mimic humans’ dietary fat consumption, we used a high fat diet containing 45% calories from fat (22% calories from saturated fat). Our data demonstrated an increase in hepatic triglycerides levels and the presence of lipid droplets within hepatocytes of mice fed HFD. Despite not worsening lipid accumulation, chronic CS accentuated the impacts of HFD on the liver suggested by the emergence of inflammatory and fibrotic markers which are indicative of NASH [25].

NASH is a progressive disease of the liver, and according to the prevailing model of the ‘multi-hit hypothesis’, lipid accumulation within the hepatocytes constitutes the ‘first hit’. On this note, insulin resistance has been demonstrated to be a key driver for the development of hepatic steatosis [24]. The ‘first hit’ increases the vulnerability of the liver to additional insults or ‘hits’ to cause hepatic injury, inflammation and fibrosis. These additional insults include increased oxidative stress, lipid peroxidation, mitochondrial dysfunction, lipotoxicity from ectopic accumulation, inflammatory cytokines/adipokines imbalance, hepatic accumulation of cholesterol, lipopolysaccharide derived from gut and the activation of innate immunity [26]. On this note, CS has been demonstrated to be an abundance source of oxidant molecules, and it is estimated that each puff of CS contains 10^14^ free radicals [27] which is believed to play a major role in the development of smoke-related lung disease such as chronic obstructive pulmonary disease [28]. However, the direct impact of CS in extra-pulmonary tissues, such as the liver is much less understood. The present study showed that CS caused liver inflammatory changes, that is most evident when CS was administered in conjunction with HFD. Excessive lipid accumulation, inflammation and injury not only marks the progression of MAFLD from simple steatosis, a relatively benign and reversible state, to the necro-inflammatory state of NASH. It is generally understood that excessive lipid flux and increased *de novo* lipogenesis by obesity may promote hepatocyte injury via substrate-overload [29]. Our data revealed that obesity-driven steatosis alone did not result in any detectable levels of hepatocyte injury, despite a significant increase in F4/80 positive macrophages. This suggests substrate overload is unlikely to be a sole culprit behind NASH which is consistent with the ‘multi-hit hypothesis’.

CS has been demonstrated to cause a variety of adverse effects in organs that have no direct contact with the smoke itself, such as liver [30]. In the present study, CS-exposure *per se* causes moderate oxidative stress in the liver in normal mice but the oxidative stress was substantially manifested in HFD-fed mice in an additive (such as 3-NT and Gpx1) or even synergistic manner (MDA content and carbonated protein and HO-1 expression). Interestingly, liver oxidative stress induced by CS and HFD individually or in combination was accompanied by similar patterns of additive or synergistic manifestation in the markers of both inflammation (IL1β, NFKβ, IKβ, F4/80 and Cd68) and fibrosis (Sirius red staining, Col4α1, Tgfβ and αSMA). These data together indicate that whereas the effect of CS on MAFLD may be mild or moderate, it has additive or synergistic effects with HFD to dramatically progress towards NASH with oxidative stress as a possible major driving factor. When the increased hepatic oxidative stress was prevented with the antioxidant tempol, the progress of NASH produced by the combined effects of CS and HFD was completely averted despite the presence of hepatosteatosis. These data confirm oxidative stress as another ‘hit’ to drive the progression of hepatosteatosis to NASH in smoked HFD fed mice.

Oxidative stress is the imbalance between the productions of reactive oxygen species (ROS)/reactive nitrogen species (RNS) generated and their removal by cellular antioxidants including GPx and other nonenzymatic molecules such as glutathione (GSH) [31]. To investigate the mechanism underlying the additive effects of smoking and HFD on hepatic oxidative stress, we first examined the expression of the key enzymes NOX4 (which produces H_2_O_2_, increasing mitochondrial ROS) [32], and Cybb the gene encoding NADPH oxidase 2 (Nox2, responsible for generating superoxide radicals (O2•-) [33] but did not detect any change in response to the combined effect of CS and HFD. Interestingly, the additive effect of CS on the increase of 3-NT and carbonylated proteins suggests a possibility of lipid peroxidation as a source of ROS for the observed hepatic oxidative stress in the presence of lipid accumulation from HFD. Additionally, the additive effect of the combined CS with HFD highly suggests that the increase in hepatic oxidative stress is attributable to a reduction in ROS removal. One of the endogenous sources for the production of ROS is mitochondria [34], and our previous studies have found mitochondrial dysfunction by HFD [6]. As CS has also been shown to damage mitochondria, further studies are needed to investigate whether CS and HFD may additively impair mitochondrial oxidative phosphorylation to exacerbate ROS production.

Although not in direct contact, CS exposure has been shown to give rise to intermediate chemicals with cytotoxic potentials. For example, nicotine is a major component of CS which is primarily metabolised by the liver. Furthermore, once inside the body, nicotine may be oxidized into cotinine which has a longer half-life has been shown to increase cellular ROS production by causing mitochondrial dysfunction [35], while depleting hepatic GSH content and catalase activity, thereby promoting oxidative injuries of the liver [36]. Hepatocyte injury in turn provokes a regenerative response in which immune cells are activated locally or recruited from the bloodstream, the extracellular matrix (ECM) is remodelled, together with hepatocyte proliferation to compensate for the lost functional hepatic tissue [1].

However when coupled with CS, long term overnutrition seen in obesity may trigger repeated hepatic injury/regeneration which may lead to the dysregulated activation of hepatic stellate cells and a ECM remodelling, resulting in the over production of collagen I and extensive accumulation of ECM leading to formation of scar tissues (i.e. fibrosis) that are disruptive to liver function [37]. On this note, elevated oxidative stress has been shown to promote liver fibrosis by stimulating the generation of collagen from the activated hepatic stellate cells and release of other profibrotic (TGF-β, α-SMA), chemotaxis (MCP-1) and proinflammatory (IL-1β, TNF-α) mediators [38]. In agreement with this, we observed both overproduction and deposition of collagen, along with augmented expressions of the relevant fibrotic mediators in the liver of the HFD-S mice. In male Wistar rats, Watanabe et al*.* [36] reported that lipid peroxidation was elevated by 2.75-fold in the liver following CS-exposure but not in the lungs. This suggests not only that oxidative stress may be distinctively driven in these organs but also a possibility that hepatocellular injury may be originated from lipid peroxidation. In line with this, our study found that hepatic MDA level is significantly elevated only under HFD plus smoking condition. Intriguingly, administration of a ROS scavenger—tempol was effective in ameliorating inflammatory and fibrotic markers in liver, suggesting lipid peroxidation is likely to be to be a major culprit underlying the hepatotoxicity observed in these mice.

In addition to nicotine and/or its intermediates, increased carboxyhaemoglobin and decreased oxygen availability may indirectly cause oxidative stress in hepatocytes. Our laboratory [39] and others [40] have demonstrated that CS-exposure increases carboxyhaemoglobin concentration to >5%, a phenomenon known as carbon monoxide toxicity. Of interest, the hepatic expression of *HO-1*, rate-limiting enzyme for the degradation of heme [41], was significantly elevated in response to concurrent CS and HFD in our study, suggesting increased iron catabolism might be at play. Future studies should focus on the role of hepatic iron metabolism, particularly in the context of CS. In addition, induction of HO-1 has been suggested to be a critical adaptive response of the liver against oxidative damage elicited by lipid peroxidation [41]. This implies that lipid peroxidation may be another important source of oxidative stress observed during concurrent CS and HFD feeding.

Tempol is a cell-permeable small molecule that scavenges superoxide (O_2_^−^) radicals, increase the rate of breakdown of the O_2_^−^ radicals into oxygen or hydrogen peroxide, mimicking the action of superoxide dismutase (SOD) [42,43]. The observation that tempol administration was able to fully antagonise the CS-induced markers of oxidative stress, hepatocellular inflammation and the resultant fibrotic response implies that targeting oxidative stress maybe a feasible approach to halt or slow down the progression of MAFLD. However, the benefits of antioxidant supplements for patients with MAFLD remain unknown due to insufficient evidence to either support or refute such therapy [44]. Presently, tempol has been used only in human subjects as a topical agent to prevent radiation-induced skin damage which appeared to be effective and well-tolerated [45]. On this note, tempol has been demonstrated to selectively modulates the gut microbiota composition and metabolism under HFD conditions [42], which may provide additional benefits under conditions of obesity. In the present study, tempol intervention was used to further support our hypothesis that oxidative stress is the main cause of NASH development (demonstrated by increased hepatic pro-inflammatory markers) and a great burden on the liver in the combined group.

**Figure 8:**
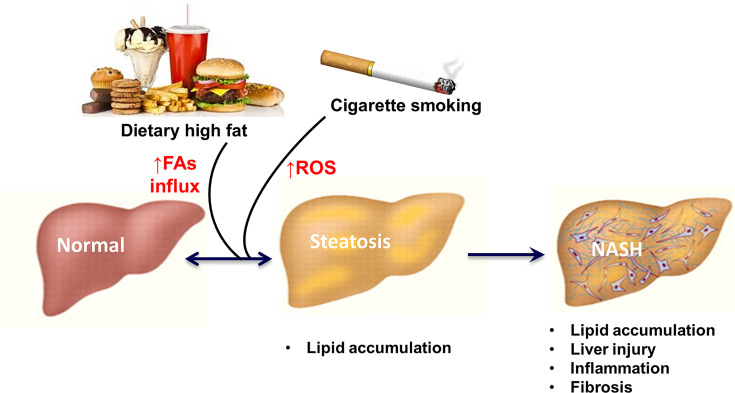
Proposed two-hit mechanism for the development of NASH Dietary high fat (HFD) causes a net influx of dietary lipids (free fatty acids) into the liver, leading to the onset of steatosis. Cigarette smoking has additive or even synergistic effect with HFD to exacerbate liver oxidative stress to drive the progression of MALFD toward NASH. The oxidative stress arising from cigarette smoke exposure constitutes a secondary insult which aggravates hepatic steatosis (but not normal liver), resulting in inflammatory and fibrotic changes as seen in NASH.

In summary, the present study shows that CS is an important source of hepatic oxidative stress, which has additive or event synergistic effect with HFD to progresses simple hepatosteatosis to steatohepatitis, setting a stage for the development of more severe and irreversible liver diseases such as cirrhosis, and hepatocellular carcinoma (Figure 8). The reduction of oxidative stress by antioxidants prevented the harmful impacts of CS on NASH pathology provides a strong proof-of-concept information that targeting hepatic oxidative stress may be an attractive therapeutic strategy.

## Clinical perspectives

- Consumption of diet rich in fat and cigarette smoking (CS) are independent risk factors of non-alcoholic steatohepatitis (NASH) and they often occur together in some populations. The present study investigated the mechanisms for the effects of high-fat diet (HFD) and cigarette smoking (CS), individually and in combination, on the pathogenesis of NASH.
- Whereas neither CS nor HFD alone result in NASH, in combination they induce typical NASH associated with severe oxidative stress in liver. Inhibition of liver oxidative stress alleviates most NASH phenotypes produced by the combination of CS and HFD except for steatosis.
- Our findings suggest that oxidative stress is a key mechanism underlying CS-promoted progression of simple hepatosteatosis to NASH. Targeting hepatic oxidative stress may be a viable strategy in halting the progression of MAFLD.

## Supplementary Material

Supplementary Materials

## Data Availability

The authors confirm that the data supporting the findings of this study are available within the article and/or its supplementary materials. Raw data were generated at RMIT University. Derived data supporting the findings of this study are available from the corresponding author J.M.Y. on request.

## Abbreviations

3NT: 3-nitrotyrosine
ALT: alanine aminotransferase
ANOVA: analysis of variance
AUC: area under the curve
CAT: catalase
CH: chow
CS: cigarette smoking
ECM: extracellular matrix
EDTA: ethylenediaminetetraacetic acid
ELISA: enzyme-linked immunosorbent assay
FBS: fetal bovine serum
FFA: free fatty acid
GPx: glutathione peroxidase
GSH: glutathione
GTT: glucose tolerance test
HFD: high-fat diet
IHC: immunohistochemistry
ITT: insulin tolerance test
MAFLD: metabolic associated fatty liver disease
MDA: malondialdehyde
mRNA: messenger RNA
NASH: non-alcoholic steatohepatitis
PCR: polymerase chain reaction
RNS: reactive nitrogen species
ROS: reactive oxygen species
SEM: standard error of mean
SOD: superoxide dismutase
TG: triglyceride
