# Supplementary material for: Exposure to cigarette smoke precipitates simple hepatosteatosis to NASH in high-fat diet fed mice by inducing oxidative stress
Source: Clin Sci (Lond). 2021 Sep 6;135(17):2103–19. doi: 10.1042/CS20210628 (PMC8436265; doi:10.1042/CS20210628)
Supplement: Supplementary Materials [file CS-2021-0628_supp.pdf]

Supplementary Data

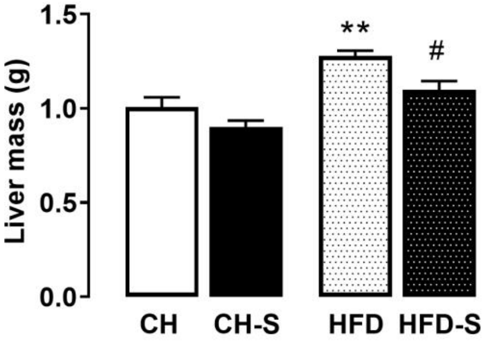

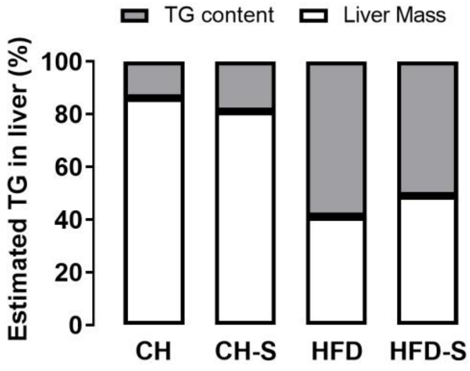

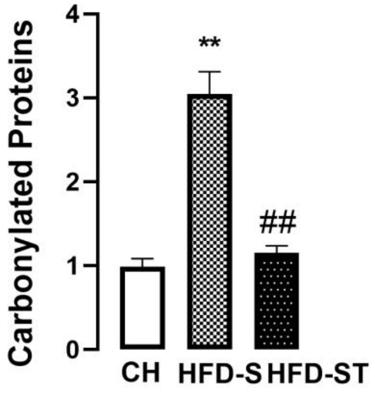

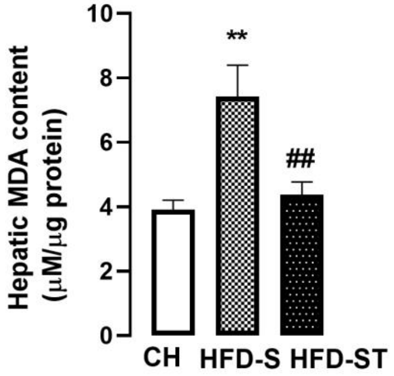

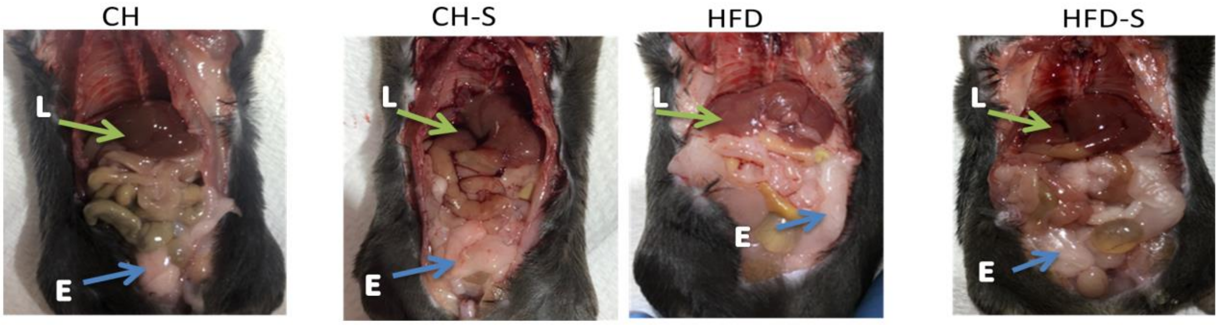

Ventral view of liver and epididymal fat pads from CH, CH-S, HFD, and HFD-S mice. (E) Epidydimal fat, (L) Liver as indicated with blue arrow and green arrow respectively.

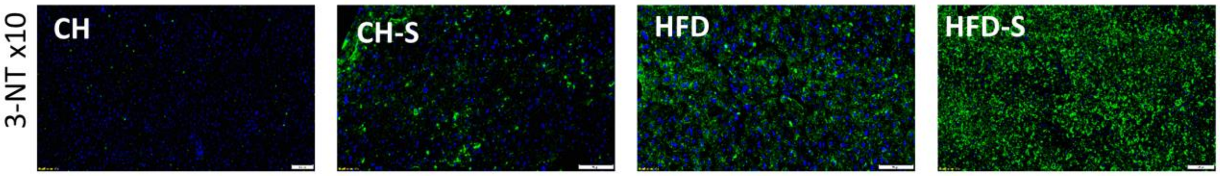

3-NT staining at 10x magnification. Smoke alone induced an increase in 3-NT expression along with HFD-feeding. The combined group induced a much higher rise in expression of 3-NT consistent with macrophage infiltration was markedly increased in this mice group compared to HFD alone.
